# Views on climate change, climate action and mental health, in young people with and without existing depression symptoms: A qualitative study

**DOI:** 10.1016/j.joclim.2025.100606

**Published:** 2025-12-24

**Authors:** M. Siyabend Kaya, Ed Hawkins, Ciara McCabe

**Affiliations:** a Faculty of Humanities and Social Sciences, Psychology Department, Abdullah Gül University, Kayseri, Turkey; b National Centre for Atmospheric Science, Department of Meteorology, University of Reading, Reading, United Kingdom; c School of Psychology and Clinical Language Sciences, University of Reading, Reading, RG6 7BE, United Kingdom

**Keywords:** Climate change, Mental health, Climate anxiety, Climate depression, Climate action, Climate messaging

## Abstract

**Background:**

Youth mental health is in crisis. Climate change has the potential to tip more young people into depression and anxiety. Knowing how young people with and without depression symptoms view climate change could guide interventions to mitigate against climate induced mental health issues.

**Materials and Methods:**

We carried out in-depth, semi-structured interviews with (*N* = 27) young people aged 18–25 (M_age_ = 20.3 years). Participants were grouped as healthy controls (C, *N* = 16, < 16 score on Mood and Feelings Questionnaire, MFQ) or had high depression symptoms (HD, *N* = 11, ≥ 27, MFQ). Using thematic analysis, we explored participants views on climate change, climate action, climate messaging, climate agency and mental health.

**Results:**

From the interviews, eight key themes emerged: (1) Negative environmental events – Climate change was understood as ranging from weather changes to natural disasters. (2) Mental health impacts – Most participants reported increased anxiety and depression, with the HD group being more pessimistic about climate change prevention. (3) Benefits of action – Focus on individual efforts. (4) Non-disruptive vs. disruptive actions – Preference for non-disruptive solutions. (5) Hope and Fear in climate messaging – balance is needed. (6) Local and global action – Emphasis on combining both approaches. (7) Leadership – Responsibility placed on politicians, institutions, and environmentalists. (8) Shared responsibility – Families, educators, governments, and celebrities all have a role in climate action.

**Conclusion:**

These findings offer valuable insights into the perspectives of young people with and without existing symptoms of depression. Notably, identifying differences—such as varying levels of climate pessimism—based on depression status highlights the importance of climate communication strategies that not only effectively address climate change but also safeguard youth mental health. This is important as those with existing depression symptoms may be more vulnerable to the psychological impacts of climate change.

## Highlights

• Young people describe the negative impacts of climate change on both the environment and their mental health.
• Young people with high depression symptoms describe feeling pessimistic about climate change and action.
• Young people think a balance of Hope and Fear in climate messaging is needed to spur action.

## 1 Introduction

Depression is one of the world’s most disabling mental disorders [1,2]. Young people are at high risk of depression [3], with over 40 % of individuals experiencing their first episode of clinical depression before the age of 20 [4]. Thus, mental health prevention and early interventions are priorities.

Climate change is the greatest global health challenge of the 21st century with the Intergovernmental Panel on Climate Change (IPCC) recently reporting that increased global heating will have catastrophic consequences for human health including increased extreme weather events and disruptions to food and water resources [5]. The physical effects of climate change on health are rapidly emerging but less is known about its psychological effects [6]. Recent reviews of qualitative studies on climate change find that people worry about threats to livelihood, worry for future generations, worry about apocalyptic futures, anxiety at the lack of response to climate change and feel helpless and disempowered [7].

As young people are most concerned and will be most affected by climate change, studies report high levels of climate anxiety (or eco-anxiety) [8,9]. Studies also find that climate change predicts future anxiety [10] and some individuals have started seeking psychotherapy for emotional problems related to climate change [11]. As anxiety and depression can be bidirectional [12] climate anxiety could precipitate new depression symptoms and worsen others [8,13]. Interestingly, studies also show that greater climate anger can reduce daily depression symptoms [14], suggesting a complex interplay between feelings about climate change and mental health. Yet how young people with existing depression symptoms view climate change and it impacts on their mental health is not clear.

Young people see adults failing to take sufficient urgent climate action [15,16], despite the data showing that action is one way to mitigate against the effects of climate change [7] and studies suggest that strong emotional feelings about climate change can initiate action [17]. However, for some who feel dismissed, criminalised, pathologized and patronised for their feelings on climate change [15] they might manage their climate anxiety by avoiding thinking about it [7]. This could exacerbate depression where avoidance characterises the disorder [18]. Further, as depression is associated with low motivation [19,20] and associated with reduced prosocial behaviours [21] this might make climate action difficult for those feeling depressed. Therefore, it is imperative to understand young people’s views of climate change and climate action with qualitative studies [22] especially in those with existing mental health conditions such as depression.

Studies suggest that fostering support from others, adapting to climate change and trying to be hopeful about the future, despite feeling loss and despair, could mitigate against negative psychological impacts of climate change [7]. Similarly, a recent review of 23 studies in young people found that messages about climate change that are hopeful, that encourage involvement in climate action, and that promote climate justice and advocacy could potentially mitigate against any psychological impacts [23]. Further, as communication and social marketing can influence population health and environmental outcomes it is important to understand the relationships between depression symptoms, climate change and climate messaging to effectively harvest this potential to combat climate change [24].

Agency, the human capacity to exercise free will, is thought a key driver of social tipping points and climate action [25]. Studies show that young people feel a lack of agency over their own actions and the structural changes needed in society [16]. Yet how depression symptoms might affect views on climate change messaging, as far as we are aware, is unknown. Therefore, the aim of this study was to examine, for the first time, in young people with and without existing depression symptoms their views on climate change, climate action, climate messaging, climate agency and mental health. Exploring these perspectives could inform more supportive climate communication, mental health interventions, and youth engagement strategies that promote both psychological wellbeing and constructive climate action.

## 2 Methods

### 2.1 Participants

Twenty-seven young people between the ages of 18 and 25 years were recruited from the UK university student population via online advertisements. We also contacted those who have given consent in previous studies on depression symptoms to be contacted about our work. Based on the Mood and Feeling Questionnaire (MFQ), we recruited those with high depression symptoms (HD, ≥27 on MFQ) and low depression symptoms i.e. controls (C, <16 on MFQ). Participants were reimbursed £30 for their time. We complied with the Helsinki Declaration of 1975 (revised 2008) and gained approval from the University of Reading Human Ethics Committee (REC reference number: 2023–003-CM). Participants received a debrief form, with contact details for The Samaritans, a registered charity that provides emotional support to anyone in distress, struggling or at risk of suicide through various services. Participants were also advised if concerned about their mood to contact their GP.

### 2.2 Procedure

After reading the information sheet online, participants had time to ask questions via email and before giving written consent on the online form. Subsequently, they completed demographic questions and the following questionnaires.

### 2.3 Questionnaires

#### 2.3.1 Depression

We used the Mood and Feelings questionnaire (MFQ) a widely used self-report tool assessing emotional, cognitive, and behavioural symptoms of depression that has been validated in large, clinically and demographically diverse samples of youth from both clinic and non-clinic settings [26], young adults in the general population [27] and young adults seeking help for their mood [28]. Items are rated on a 3-point Likert scale (0–2), yielding scores from 0 to 66, with higher scores indicating greater severity. A suggested cut-off score of 27 has been shown to be optimal, offering the greatest diagnostic confidence when distinguishing clinical from non-clinical populations, based on the intersection point of sensitivity [.78 (95 % CI, 0.67 to 0.89)] and specificity [.78 (95 % CI, 0.66 to 0.89)] [29].

#### 2.3.2 Eco-Anxiety

The Hogg Eco-Anxiety Scale [13] comprises 13 items about the preceding two weeks and frequency with which one experienced environmental anxiety. A higher score indicates higher eco-anxiety, with the potential score range from 0 to 39. The scale demonstrates excellent internal reliability ( = 0.92) [13].

#### 2.3.3 Pro environmental behaviours

For pro-environmental behaviours, we utilized a survey adapted from [14]. This survey comprised a total of sixteen items, with eight focusing on individual behaviours (e.g., recycling and composting) and the remaining eight pertaining to collective behaviours (e.g., participating in protests). Participants specified their level of participation as a percentage over the last year, choosing from a scale spanning ten values, ranging from 0 % to 100 %. Higher scores indicate a greater number of pro-environmental behaviours performed over the past year.

### 2.4 Semi-structured interviews

Using their research expertise in the fields of youth mental health and climate change, the authors created a semi-structured interview (Table 1). Interviews were conducted online (Microsoft Teams ∼35 min (range: 17–56 min). Data saturation was reached when no new information was observed. Interviews were audio-recorded, transcribed verbatim, checked for accuracy, and subjected to thematic coding. Field notes were made after the interview and used to aid analysis. We employed Thematic Analysis (TA) [30] similar to our previous studies [31]; see supplement for more details on TA.

**Table 1:** Semi-structured Interview guide.

**Question Number**	**Question Text**
1	What does climate change mean to you? and how does it make you feel?
2	Are you actively trying to prevent climate change? If yes, how? And if not, why not?
3	What do others think about climate change? Any examples
4	What are others doing about it? Any examples?
5	What do you think are the best ways to make people care about climate change? And why?
6	What do you think are the best ways to make people take action on climate change? And why?
7	Do you think it matters who the climate change message comes from? And why? Any example?
8	Would who the message comes from make a difference to your action? And why? Any example?
9	Do you feel you have any control over climate change and its consequences?
10	Who do you think is in control of climate change and action? (cultural, political?)
11	Does climate change affect your mental health? How and why?

### 2.5 Thematic analysis

We employed Thematic analysis (TA) to identify and analyse data patterns of meaning useful when investigating how a group conceptualizes a particular phenomenon [30]. TA is not tied to a particular ontological or epistemological position, therefore, the researchers adopted a post-positivist critical realist stance [32,30]. Furthermore, to align this work with emerging literature on climate change experienced by people with mental health problems, we chose a pragmatist approach and an abductive process of analysis [33]. Without first attempting to fit the data into pre-existing coding schemes, the data were examined, and we did not dismiss possible themes that did not fit current literature, nor did we identify themes unless they were evident in the data.

We considered our own sources of bias and prior assumptions, including knowledge of depression and climate change and climate action. The data were analysed using constant comparative techniques based on Braun and Clarke's six-stage TA method [34]. In stage 1), the first author familiarized themselves with the data by conducting and transcribing interviews and then re-reading the transcripts. In stage 2), line-by-line coding was conducted. The process of coding was inductive and iterative, with constant comparisons between and within transcripts. Initially, all data were coded for both explicit and implicit meanings. The labelling of codes focused on capturing subjective experiences and viewpoints [35,36]. In stage 3), codes were combined into potential themes that reflected the data's major characteristics and patterns. In stages 4) and 5), themes were evaluated by examining all codes and themes in aggregate [34,37]. Tentative themes were reviewed by the research team and during these coding meetings, alternative interpretations of patterns in the data were considered and discussed until a consensus was reached. In the final stage 6), themes were finalized, and quotations exemplifying each theme were identified.

## 3 Results

Of the total participants (*N* = 27, mean age 20.3 yrs), 11 participants had high depression symptoms (HD ≥ 27 on MFQ) and 16 participants were controls (< 16 on MFQ) (see Table 2 for demographics). Participants who had high scores on depression also reported twice as much eco-anxiety as the controls., but this was not significant using an independent samples *t*-test (t(23), = −1.38, *p* = 0.09), possibly due to the small sample size not suitable for quantitative analysis.

**Table 2:** Participative descriptive statistics and demographic characteristics.

**Descriptives**	**Controls**	**High Depression Symptoms**
Age (Mean, SD)	22.07 (0.61)	21.09 (0.53)
Female	13	8
Male	3	3
White	12	6
BAME	4	5
Depression (MFQ) (Mean, SD)	14.29 (2.19)	38.55 (2.46)
Eco-Anxiety (Eco-A) (Mean, SD)	4.43 (1.40)	8.64 (2.95)
Pro-Environmental Behaviours (PEB)	33.35 % (3.79 %)	44.35 % (7.47 %)

Young people’s views emerged in eight themes. We show examples from those with high depression symptoms (HD) and controls (C) below. For additional quotes on each theme please see the supplemental information document.

### 3.1 Negative environmental events

**Fig. 1:**
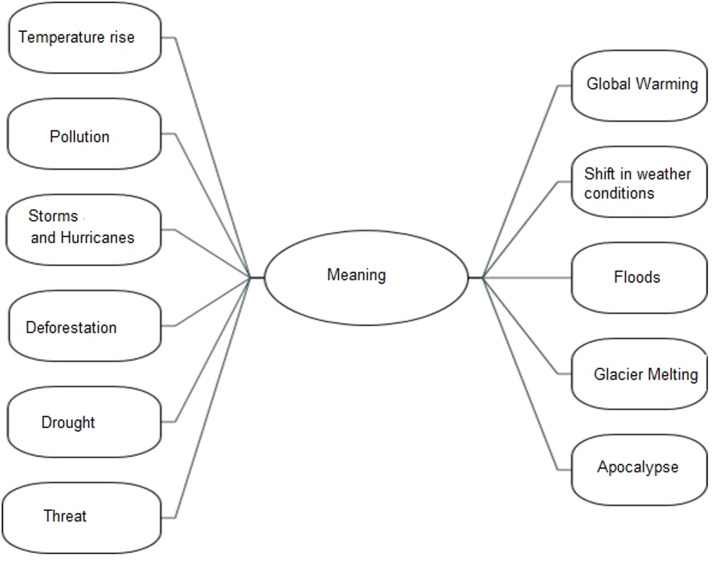
Negative environmental events.

Participants defined climate change as a negative situation that lasts for many years, ranging from changing weather conditions to natural disasters (see Fig. 1).

*“I link it closely with global warming and I associate it with like theorising global temperature and with that comes like rising sea levels and stuff like that……” 02C*

*“If I could draw a picture about the climate change it would be like really bad weather like fire, and you know storms and like all sorts of big waves and three, no trees…… and it would be like an apocalypse scene……” 05C*

### 3.2 Negative impacts on mental health

**Fig. 2:**
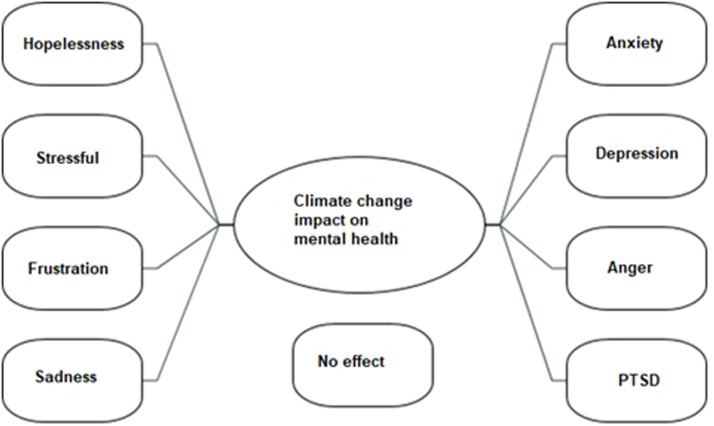
Negative impacts on mental health.

Almost all participants expressed that climate change affects their mental health, especially with feelings of depression and anxiety (see Fig. 2).

*“it makes me feel like really, really upset and really terrified and sometimes it makes me feel almost helpless. So feel I am angry, because it feels like it's out of my control as an individual also I'm thinking about people, but then I also think about animals, and you know the animals they don't have a voice so, there are like devastating videos and images of animals in forest fires and floods and they are the ones that can't speak up and that's what makes me really upset……” 03HD*

*“…… I try not to think about things like that (climate change), but if I do think about it, it does affect my mental health. I just don't like to think about the people in the future having…… because of what's happening today, they can't live their best life, because we've taken everything from them. I feel definitely stressed and anxious, but also quite depressed, like what's the point if it's all gonna be really bad in the future? What's the point of going on now? That's really terrible, but it's kind of, you know like having children in the future. I think people are gonna start wanting to have less children because the world that they're gonna be born into is just not very nice……” 05C*

The expressions of HD and controls differed qualitatively in this theme in that the HDs were more pessimistic than controls about the prevention of climate change. Here are some examples:

*“……When you check the statistics and you see that the majority of like climate change is being caused by more celebrities, more rich people and private jets and things like that, these sort of protests are a completely pointless and be completely counterproductive to the goal, which is why I'm very pessimistic about climate change because I look around me and I see that nothing is really truly being done in a proper way that would be effective……” 13HD*

*“……there's just disasters everywhere. Just because of climate change and you're seeing irreversible damage. Now they're saying there's rivers that are drying up. And by 2050, there's some crazy stats that, yeah, even if we stop driving our cars right now and we stop our individual pollution, there's so much damage that has been done. Even by 2050, there's gonna be irreversible stuff……” 02HD*

The following excerpt describes how acknowledging climate change could negatively impact mental health in a HD participant:

*“……I see that some people around me just completely dislike and don't pay attention to climate change, and this kind of causes their mental health to be better, I think. It is like "the kind of ignorance is bliss"……..” 05HD*

The same participant also expresses anger and frustration at the lack of action of others:

*“We've known that something was happening with the world for so long but people with a lot of money have decided to destroy the world and kind of try and force everyone to ignore the problem and it's got to the point where we're at breaking point in if we don't do anything now, then ……the world's just gonna fail. But I'm angry at that because we could have done something much sooner ……” 05HD*

Some participants also mentioned how climate change could lead to despair, trauma and even pre and post-traumatic stress disorder, examples:

*"So I would say that of course extreme weather events and mental health are related. As a result, many people will experience high levels of psychological distress, let's say….and some may develop some serious mental health problems such as PTSD, depression and stuff like that….the concerns we have about the future of the planet and the impacts of climate change that can lead to feelings of helplessness and despair. This can contribute to anxiety which can make it more difficult to engage in enjoyable activities.”. 03C.*

*"if you're stable, you can contribute to the whole idea of preventing the climate change happens, but if you're in a bad mood like you feeling depressed, you have a PTSD, let's say, which reminds you of some experiences you had with climate change, uh, you may not be able to contribute as much.” 03C*

*"Obviously, there are direct impacts of climate change like psychological distress and trauma……as events are happening in the world, like with weather and stuff and then obviously there is indirect ones being the threat of the future climate related disasters.” 17C*

### 3.3 Benefits of climate action

**Fig. 3:**
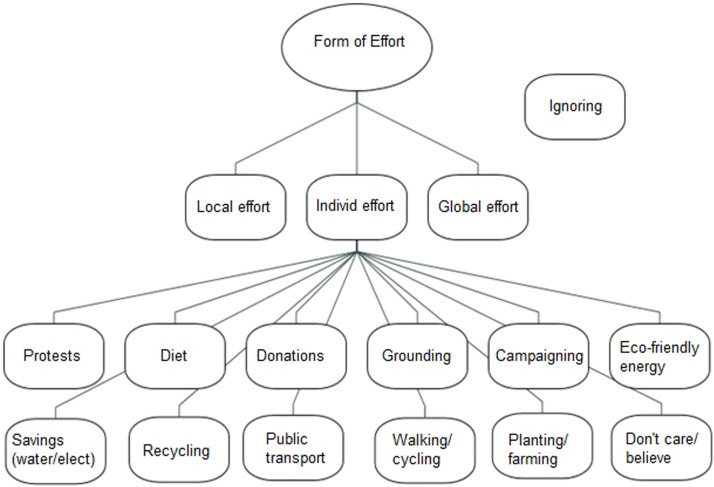
Benefits of climate action.

Participants describe forms of climate action (Fig. 3) especially individual effort.

The statements below illustrate the sub-themes of individual effort:

*“So personally…. I keep recycling stuff, keeping my electricity down as possible, not wasting water, using public transport, and I am also subscribed to a Greek website which sends these emails about the news around the world, and I usually make donations there about like climate change stuff and animal issues……” 03C*

*“I try to eat plant based as possible. I don't buy plastic water bottles, I reuse my cups and I recycle as best as I can and I don't use energy that's not necessary and I sign a lot of petitions, try and spread some awareness by sharing those petitions and everything to try and cause the laws to change in order to make a bigger effect on the world.” 05HD*

Some participants also describe not being able to connect with climate change impacts far away:

*“……let's say the Pakistan floodings, right and so, my friends will bring it up like, uh, have you heard the news? It's like, yeah, this is terrible, but it doesn't…. I think it's the feeling. It's maybe it's a current Gen. thing as well. Most of my friends are around the similar age and so, it's not like me driving around in the car is going to make a difference. You know what I mean!” 02HD*

### 3.4 Non-disruptive vs disruptive action

**Fig. 4:**
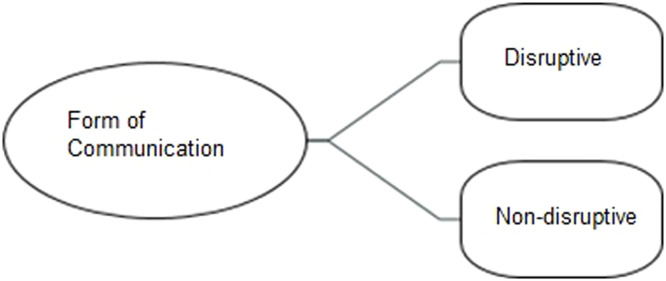
Non-disruptive vs disruptive action.

When asked about climate change messaging the young people described two main themes: disruptive and non-disruptive (see Fig. 4).

The majority of the participants agreed that the methods to highlight the need for climate action should be non-disruptive.

*“I don't agree with like destroying public property or like expensive things …… I think peaceful protests are the most effective……” 15HD*

*“I don't really like those sorts of things (disruptive action by activists towards national galleries etc.) … I think it takes away from the point. Because I think that's just sort of like defacing artwork. People talk more about that than the actual cause for the thing that they did………” 05C*

*“If you are however disrupting work for a fossil fuel company, for example, if you're doing some sort of lockdown or something that's disrupting the actual functioning of these corporations in a way that makes it clear to them that their operations are being threatened….. If they destroyed a work of art that had nothing to do with the problem they're protesting, I think that's a shame and also, I think it discredits people who are genuinely trying to bring awareness to the situation………” 04HD*

Further, some participants thought a mix of disruptive and non-disruptive methods was a good idea:

*“I looked a lot into those actions (throwing soup at Van Gogh painting) especially like with the artworks and people were so outraged …. I understand why they are doing it because it obviously did bring attention to the issue and it was something that you could see all around the world…but I think as long as there's no actual harm being done, I personally wouldn't do it myself, but I'm also not holding it against those actions either……” 06C*

### 3.5 Hope and fear as emotional motivators

**Fig. 5:**
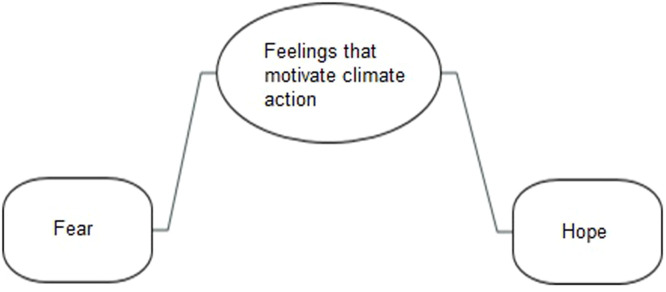
Hope and fear as emotional motivators.

There were some mixed views on emotional motivators for climate messaging (Fig. 5). Some HD stressed the need to employ the “fear” factor in climate change messaging, while some of the controls emphasised “hope” as a better motivator tb be used in climate messaging. Both HD and C mentioned a balance between the two could be the best approach.

Here are some examples from HD participants showing their feelings about what message might best motivate climate action:

*“……I would say fear is more helpful because if we properly, don't start making an impact then hope isn't really there anymore because it's all contributing really quickly……” 15HD*

*“If the message is hope, they probably feel like there is already hope in like things are already getting better. They probably feel less inclined to make changes themselves. If it's fear, then they're probably more likely to be driven to change things” 01HD*

An example of a control participant describing the usefulness of positive messaging over fear:

*“…… I think a lot of the news is really negative…and if we were given a little bit more positive stuff that might encourage us to keep going, because we can see the benefits rather than always seeing the bad stuff……, because then it's like, oh well, I'm trying really hard, but all I see is the bad stuff. So why do I keep trying? if nothing's, you know, nothing's getting better from what I'm doing?” 05C*

Other participants thought a mix of both fear and hope in messages of climate change is needed:

*“……I think people don't understand the gravity of the situation unless they're afraid of the consequences, if not for their own lives, then for the future generations. But all kind of, I mean, all activism is kind of useless if you don't have hope. So, I think fear and hope, both are required to awaken people……” 04HD*

*"I'd say both (fear and hope). I mean, it's hard to say because it can feel quite hopeless if you make someone so fearful of climate change, they won't do anything, because then they're too fearful and hopeless. So, I think it would be a mix (of fear and hope). 05C"*

### 3.6 Local & global action is needed

**Fig. 6:**
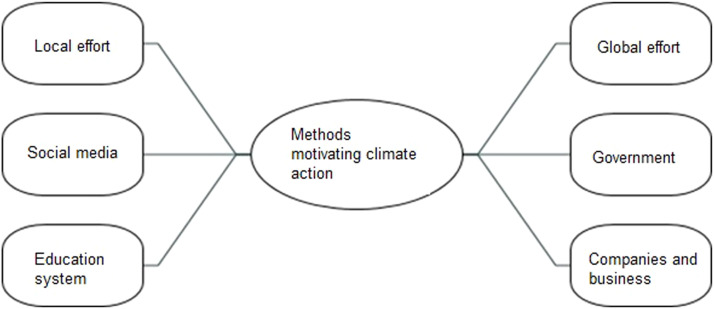
Local & global action needed.

Although the participants discussed a variety of ways to encourage people to take climate action (see Fig. 6), they mostly emphasised the value of fusing local and global initiatives:

*“I think local action, smaller communities and the people around you is what really makes you think maybe more deeply about your actions and how you could maybe change or become more aware, but obviously global climate change is a global issue and it's very important to also tackle it globally. So I think both are really important………” 06C*

*“I think global would have a greater impact, but I think they have to start locally first before we can start increasing globally. So like a bottom up approach might be more useful……” 13HD*

### 3.7 Leadership in climate action

**Fig. 7:**
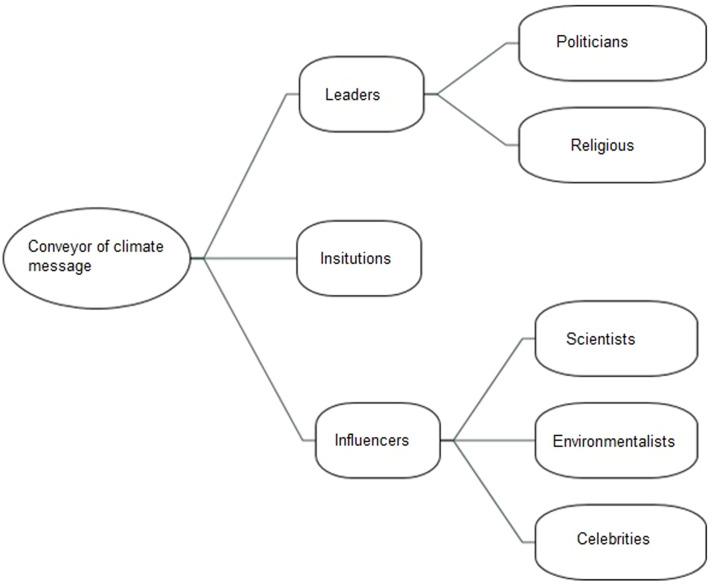
Leadership in climate action.

When asking young people about who is best to communicate climate change three sub-themes emerged: Politicians, institutions/companies and environmentalists (see Fig. 7.)

The following is an example of the role of science in communicating climate change:

*“When I saw the climate stripes, the one that goes from blue to red, that's when I really understood how critical the stage we are at right now. Because what you're looking at, statistics over several decades, to see how it's changed and to see that it's the same around the world is a little…… I think that is what would push me to think about what we should do. We just need to start doing something immediately……” 04C*

Participants also felt that although companies can be effective in conveying a climate change message, they are not sincere in doing so:

*“Corporations do things to seem like they're being eco-friendly, but it's just for business. Umm, so I know that Coca-Cola started doing a thing where the caps on their bottles are attached so that it's easier to recycle them but it feels a bit gimmicky and like they're just doing it to seem like they're helping when in fact, if they really cared, I think they could make bigger changes……” 11HD*

### 3.8 Universal responsibility

**Fig. 8:**
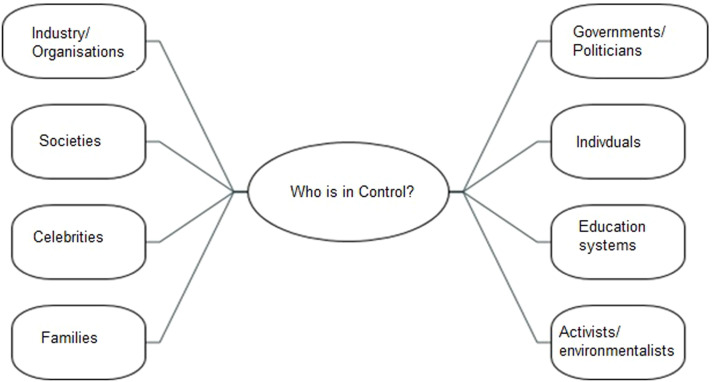
Universal responsibility.

When thinking about who is in control of climate change and action the participants thought widely about it, from families to education, to government to celebrities (see Fig. 8).

The quotations below summarise thoughts on the need for structural, policy and judicial changes regards climate action:

*“I don't think that on an individual level you can do much about it. I think that it's more of like a joint affair and a government thing and things need to be changed on a larger level in order to combat it……” 02C*

*“I think it's a mix of political figures and probably just really rich people, billionaires and people with a lot of influence and the people who own the companies that refuse to stop doing things that are affecting climate change and the people that control the media and the news and everything…. and that we need to bring in the law. The people that fund politics and the media are the people who have the most influence…. I think the politicians are kind of a way to bring beliefs forward and make change……” 05HD*

Finally, participants also stressed the importance of education:

*“I think schools have some degree of control here because if you've grown up like I have and been told that the environment is important, you do hold that it is. It's just I don't feel like I was ever told how to do anything about it. I was just told it's important……” 14HD*

For extra quotes on each theme please see the supplemental information document.

## 4 Discussion

The aim of this study was to examine for the first time in young people with and without existing depression symptoms their views on climate change, action, messaging, agency and mental health. Understanding how young people with existing depression symptoms perceive such issues is crucial because they may experience and interpret them differently from their peers. Further, climate change is thought to possibly precipitate new depression symptoms and worsen others [8,13]. Exploring young people’s perspectives could inform more supportive climate communication, mental health interventions, and youth engagement strategies that promote both psychological wellbeing and constructive climate action.

Interestingly we found that the young people with elevated depression had double the eco-anxiety scores of the control participants. This is an interesting finding but was not quantitively significant, likely due to the small sample size. Across the board however the young people recognized climate change and its harmful effects on the environment, similar to previous studies [38]. They cited physical events such as droughts, storms, and floods and compared climate change to the apocalypse, underscoring the severity with which they perceive its impact on the natural world.

The young people also described how climate change made them feel hopeless, anxious, depressed, sad and angry, in line with previous studies [[39], [40], [41], [42], [43], [44]]. They also reported how they felt that feelings of despair, being terrified and helpless caused by climate change could lead to pre- and/or post-traumatic stress disorder (PTSD). This is in line with previous studies also reporting that climate change could lead to trauma such as PTSD [45,46,39,47,48]. Pre-traumatic stress is created from fears of future threats from news about floods, storms, droughts, heat waves, wildfires, and pandemics related to climate change [45,46], while studies find that experiencing the physical effects of climate change can lead to PTSD [39,47,48]. Taken together, we show that young people experience serious negative mental health impacts such as feelings of depression and despair from climate change events and when thinking about future impending climate disasters. Thus, we support the view that climate change-related depression and depression exacerbated by the reality of climate change reflects actual, real, experiences of threat. We propose that one way to combat this is through climate action (individual, collective, political) to mitigate and better adapt to climate change.

Our findings also highlight the importance of discussing climate change and climate action, early, potentially starting in schools and preschools. Currently, there is little discussion with young people in schools about climate change and climate action; however, this is set to change in the UK as the government has laid out plans for sustainability leadership and climate action plans in education starting in 2025 Sustainability leadership and climate action plans in education - GOV.UK. However, it is not clear how much of this plan will recognise the impact of climate change on youth mental health.

Therefore, to help prevent long-term mental health issues, our results and others recommend initiating conversations on climate change and climate messaging to give young people the right information and tools to help them cope [[49], [50], [51]]. Further, psychological treatments could be tailored specifically for children and young people that are feeling anxious or depressed about climate change, to help mitigate long term mental health issues [[52], [53], [54], [55]]. For example, the UK NHS recognizes eco-therapy, a therapeutic approach that uses nature-based activities to promote mental and physical well-being, as an evidence-based way to support conditions like depression, anxiety, and stress [56,57]. Some NHS trusts and local services collaborate with charities, social prescribing programs, and community groups to offer eco-therapy sessions, where issues around climate change can be addressed [58]. Our research supports the need for such approaches and reveals how young people with existing mental health issues such as depression could have their symptoms exacerbated by climate worry. Our findings support the calls to raise awareness among practitioners and guide them in incorporating eco-therapy into their clinical practice [59].

We also found that individuals with more severe depression symptoms were more pessimistic about the future concerning climate change. They described seeing disasters everywhere and beliefs that much of the damage from climate change is already irreversible. These findings confirm the need for careful messaging of climate change so as not to exacerbate mental health issues in those already with depression symptoms. As depression is often characterized by heightened pessimism which predicts depression [60], these results highlight the increased vulnerability to further mental health issues from climate change among individuals already experiencing depression [41].

When discussing climate action, participants acknowledged the need for collective effort, but they described in more detail their own individual efforts such as recycling, using less, eating more plant-based food and using more public transport. However, they expressed worries about how individual effort might not be enough and how hard it is to stay hopeful especially when big corporations are less inclined to climate action. They also described how connecting one’s individual actions in the UK to climate change far away was difficult. Thus, these findings show that young people could be more supported in knowing what is going on regards climate action at a national or global level, to further inspire them. Further, the data suggests they should be given more information about how they could become involved in larger scale advocacy which provokes sustainable change. For example young people could be signposted to climate action groups and information websites from reputable organisations such as UNICEF [61].

The young people believed non-disruptive methods of climate messaging are best even though some disruption could bring attention to the climate issue, but it could turn people against the climate message. Although previous studies have reported a positive link between hope and climate action [[62], [63], [64], [65]] participants in our study suggested that a balance of hope and fear may be the most effective approach. To our knowledge, this is the first study to examine emotional climate messaging in young people with and without depressive symptoms. Because fear-based messages could exacerbate anxiety or depression, combining hopeful narratives with actionable climate strategies may both motivate young people and safeguard their mental health. Notably, research shows that hope is most effective when paired with self-efficacy, the belief that one can make a meaningful difference, which increases the likelihood of taking action [66]. Given that depression is often associated with low self-efficacy [67], future research could explore climate messages that integrate both hope and self-efficacy to inspire action across all young people, regardless of mental health status.

Participants described a variety of ways to encourage people to take climate action. They emphasised the value of fusing local and global initiatives to build into something more global over time. This is in line with climate governance as a decentralized multi-level bottom-up approach [68] where multiple centres of local decision-making can contribute to more effective global climate governance.

Young people emphasized that scientists, through their use of statistics, are effective communicators in conveying the seriousness of climate change whereas messaging from commercial companies could be viewed as insincere. Additionally, they felt environmentalists are particularly effective in leading climate change protests. Thus, young people value clear, fact-based credible communication and are sceptical of those who might have ulterior motives.

Finally, the young people emphasized that the responsibility for addressing climate change lies with us all, from families and educators to governments and celebrities. They stressed the need for everyone to work together, supported by government policies and judicial reforms. They reported that climate change action needs collaboration which aligns with studies showing the need for combining individual and policy-driven approaches [69].

### 4.1 Limitations

Regards limitations of the study, gender and ethnicity could have been more balanced across the participants, and therefore it is not clear if there might be different responses with more males and with more participants from different ethnic groups. Future work should also address ideas about causality and investigate if and how experiences of climate change can lead to PTSD, perhaps examining young people longitudinally who have experiences of extreme weather events.

### 4.2 Conclusion

Our findings align with previous research highlighting young people’s deep concern for the environment. We found participants emphasized the need for strong governmental action and clear, balanced, and trustworthy climate communication—prioritizing scientific information over commercial messaging. They described how climate messaging should strike a balance: acknowledging the seriousness of climate impacts without inducing hopelessness. This is especially important for youth experiencing elevated depression symptoms, who may be more vulnerable to pessimism. Given that those with depressive symptoms are at greater risk for future mental health challenges [12] our study reinforces the growing call to integrate mental health support into climate policy, both to prevent the onset of mental health conditions and to reduce the risk of worsening existing symptoms. Finally, our findings suggest that signposting young people to climate action and advocacy groups could in turn aid climate action and help mitigate the negative impacts of climate change on mental health.

## CRediT authorship contribution statement

**M. Siyabend Kaya:** Writing – review & editing, Writing – original draft, Validation, Project administration, Methodology, Investigation, Formal analysis, Data curation. **Ed Hawkins:** Writing – review & editing, Supervision, Funding acquisition, Conceptualization. **Ciara McCabe:** Writing – review & editing, Supervision, Resources, Project administration, Methodology, Funding acquisition, Conceptualization.

## Declaration of competing interest

None.

## Appendix Supplementary materials

Image, application 1
