# Supplementary material for: Views on climate change, climate action and mental health, in young people with and without existing depression symptoms: A qualitative study
Source: J Clim Chang Health. 2025 Dec 24;27:100606. doi: 10.1016/j.joclim.2025.100606 (PMC13184488; doi:10.1016/j.joclim.2025.100606)
Supplement: Supplementary file 1 [file mmc1.docx]

Supplementary doc

**Views on Climate Change, Climate Action and Mental Health, in Young People with and without Existing Depression Symptoms: A Qualitative study**

**Thematic Analysis:**

We employed Thematic analysis (TA) to identify and analyse data patterns of meaning useful when investigating how a group conceptualizes a particular phenomenon [1]. TA is not tied to a particular ontological or epistemological position, therefore, the researchers adopted a post-positivist critical realist stance [2] [1]. Furthermore, to align this work with emerging literature on climate change experienced by people with mental health problems, we chose a pragmatist approach and an abductive process of analysis [3]. Without first attempting to fit the data into pre-existing coding schemes, the data were examined and we did not dismiss possible themes that did not fit current literature, nor did we identify themes unless they were evident in the data.

We considered our own sources of bias and prior assumptions, including knowledge of depression and climate change and climate action. The data were analysed using constant comparative techniques based on Braun and Clarke's six-stage TA method [4]. In stage 1), the first author familiarized themselves with the data by conducting and transcribing interviews and then re-reading the transcripts. In stage 2), line-by-line coding was conducted. The process of coding was inductive and iterative, with constant comparisons between and within transcripts. Initially, all data were coded for both explicit and implicit meanings. The labelling of codes focused on capturing the subjective experiences and viewpoints [5, 6]. In stage 3), codes were combined into potential themes that reflected the data's major characteristics and patterns. In stages 4) and 5), themes were evaluated by examining all codes and themes in aggregate [4, 7]. Tentative themes were reviewed by the research team and during these coding meetings, alternative interpretations of patterns in the data were considered and discussed until a consensus was reached. In the final stage 6), themes were finalized, and quotations exemplifying each theme were identified.

Extra Quotes from themes:

***2-*Negative impacts on mental health,**

Almost all participants expressed that climate change affects their mental health, especially with feelings of depression and anxiety

*What's the point of going on now? That's really terrible, but it's kind of, you know like having children in the future. I think people are gonna start wanting to have less children because the world that they're gonna be born into is just not very nice...” 05C*

***3-*** **Benefits of climate action**

The quotation below shows that although individual effort is very important, it is not enough on its own.

*“I think that if every individual did their best it would that would be different but say like …reusing things that you own and stuff. It's great to do your part, but then when they're burning fossil fuels and cutting down the, you know, the forest at rates that we couldn't even imagine, that's a lot bigger. That's a huge scale. And then us doing things just individually. It just doesn't. I think it's like a drop in the in the ocean...” 05C*

*“Yeah, if everyone thinks that they can't make a difference, then no one will. So yeah, that's the thing you have to, it's a really fine line because you we do need to take personal responsibility. And I think that I do in terms of taking care of what I do and being sustainable and things like that. But it's hard to stay hopeful when you know that big companies that could be making much bigger changes in the way that they operate...” 05C*

Some participants also discussed how and why people disregard/ignore climate change:

*“Most people, keep ignoring these things. Umm, I don't know if that's a fact, but I believe that governments and in general like individuals do not show a real effort for preventing climate crisis. Umm, keep ignoring this stuff because they do not like care about the future of this planet. They may feel hopelessness, but they won't show like. They want to express their feelings towards these issues. Thats all...” 03C*

*“It seems like, it's gonna be the next generations problem. I think that's always kind of being the mentality. So it's like, yeah, I'll try and do more, but realistically, it seems like nothing to do...” 02HD*

The following statement is an example of how someone with depression symptoms feels about individual everyday climate action:

*“..Electricity with keeping stuff off right? So like this is something I've done consistently. So like let's say I turn the plug off now for the TV, right? So I'm going to sleep at night. Why do I need the switch on to the TV? You know, it's just wasting electricity. Let me turn off now in the morning when I wake up. And I go to turn on the TV, I'm getting a little bit annoyed. Agitators like Ohh, I forgot to turn on the plug. Let me turn on the plug then in another. But it's not just one device, it's several devices. Another issue for example, I stick my phone on charge, but then I have to turn the plug back on. So compared to climate change, where you can easily just kind of forget about it, you know what I mean? Because it's a bigger problem, whereas this is smaller problems that actually disturb you. You know what I mean...” 02HD*

***5-*** **Fear and hope as emotional motivators for action,**

*"I think spreading awareness needs to have that it does need to have some element of fear and danger because that's how you capture people's attention, isn't it? And that's how movements kind of work in that sense. But it would also be really good and encouraging and hopeful to see some reporting on positive things that people are doing. 13C"*

*"I think that possibly a balance of the two would be the best option because I think that if you use the destructive facts continuously then that would just, I feel like, encourage mongering.*

*But then again, if you only use the non-destructive facts then that could indicate that it's not as bad as it actually is, and so I think that using both would probably be the best option. 16C"*

***6-***. **Local & global action is needed,**

*“There has to be a certain level of the right feel like different generations you use different methods, so definitely for like the younger generation social media and things like that and kind of making people aware of what they can do to help, whether it's more sustainable living or petitions or anything....And then for the old generations, I think and I don't know. Maybe just making it more aware and their local space what they can do and having very big sign posted ways to be more sustainable. And the reason why they need to be sustainable and kind of putting it in like shops and things where they would constantly see it and be reminded that they should be trying to do that way...” 05HD*

***7-*Leadership in climate action**

Some participants thought environmentalists could have impactful climate communication:

*“..Well, I think I think protesting is really effective to get your message out... And like Greta Thunberg has become, really effective in protesting...” 15HD*

***8*** **Universal responsibility**.

*“If I thought a bunch of people were also doing little things to help make a change, then yes, that's like, you know, one soldier doesn't accomplish anything on their own...” 14HD*

*“Like I mean…if I decide to like switch off the lights….. if I if my entire action was based on kind of reducing my personal impact on climate change….even if everybody on the planet did that, I don't think it would make that much of a difference compared to massive company like fossil fuel companies, for example. So, I think collective action in terms of putting pressure on these companies, putting pressure on the implementation of laws to improve laws about, and carbon footprints, or emissions, or the use of fossil fuels or things like that. So, I think for me it would be more policy level action rather than individual interventions ...” 04HD*

1. Harper, D. and A.R. Thompson, *Qualitative research methods in mental health and psychotherapy: A guide for students and practitioners*. 2011: John Wiley & Sons.

2. Guba, E.G., & Lincoln, Y. S., *Competing paradigms in qualitative research*. Handbook of qualitative research (pp. 105–117). . 1994, In N. K. Denzin & Y. S. Lincoln (Eds.), : Sage Publications, Inc.

3. Patton, M.Q., *Qualitative research & evaluation methods*. 2002: sage.

4. Braun, V. and V. Clarke, *Using thematic analysis in psychology, .* Qualatative Research in Psychology, 2006. **3:2, 77-101,** .

5. Huggett, C., et al., *A qualitative study: experiences of stigma by people with mental health problems.* Psychology and Psychotherapy: Theory, Research and Practice, 2018. **91**(3): p. 380-397.

6. Tuckett, A.G., *Applying thematic analysis theory to practice: A researcher’s experience.* Contemporary nurse, 2005. **19**(1-2): p. 75-87.

7. Guest, G. and E. McLellan, *Distinguishing the trees from the forest: Applying cluster analysis to thematic qualitative data.* Field methods, 2003. **15**(2): p. 186-201.
